# Recent highlights and breakthroughs in immunotherapy for head and neck cancers

**DOI:** 10.1097/CCO.0000000000001211

**Published:** 2026-03-05

**Authors:** Joanna A. Vuille, Petr Szturz

**Affiliations:** Medical Oncology, Department of Oncology, University of Lausanne (UNIL) and Lausanne University Hospital (CHUV), Lausanne, Switzerland

**Keywords:** anticancer antibodies, head and neck cancers, immune checkpoint inhibitors, immunotherapy

## Abstract

**Purpose of review:**

This review highlights recent advances in immunotherapy for head and neck oncology, focusing on pivotal studies, both early-stage and late-stage, published or presented in 2025.

**Recent findings:**

Noteworthy results were reported with immune checkpoint inhibitors in mucosal squamous cell carcinoma of the head and neck (SCCHN) (KEYNOTE-689 and NIVOPOSTOP trials evaluating pembrolizumab and nivolumab, respectively, in the perioperative setting); in nasopharyngeal carcinoma (DIPPER and DIAMOND trials evaluating camrelizumab and toripalimab, respectively, in the curative setting with radiotherapy, and tagitanlimab in recurrent and/or metastatic disease); in cutaneous squamous cell carcinoma of the head and neck region (C-POST trial in the adjuvant setting and the combination of avelumab with cetuximab in the palliative setting); and in BRAF V600E-mutated anaplastic thyroid carcinoma (pembrolizumab with dabrafenib and trametinib). Passive immunotherapy targeting tumor-associated antigens also showed encouraging activity in R/M-SCCHN (petosemtamab, ficerafusp-alfa, amivantamab, enfortumab vedotin) and in heavily pretreated R/M nasopharyngeal carcinoma (antibody-drug conjugates becotatug vedotin and izalontamab brengitecan [iza-bren]).

**Summary:**

Recent advances highlight a rapid surge in positive immunotherapy trials across different head and neck cancer entities, with clinical benefit observed both when immune checkpoint inhibitors are moved earlier in the disease course and when they are combined with agents targeting resistance mechanisms or enabling more precise drug delivery to tumors.

## INTRODUCTION

Immunotherapy has become an essential pilar of oncologic treatment in the last two decades, radically changing the landscapes of some malignancies such as melanoma and pulmonary cancers. In the head and neck field as well, immunotherapy offers new treatment avenues which are being intensively studied.

The field of immunotherapy encompasses a large range of tools related to the immune system, from specific molecules to sophisticated engineered cells. It can be conceptually divided into two main groups: active or passive immunotherapy, depending on its ability to integrate and collaborate with the patient's immune system. The *modus operandi* of active immunotherapy, including immune checkpoint inhibitors (ICIs), cancer vaccines and oncolytic viruses, relies on the activation of one's immune cells, thus boosting its recognition and/or active killing of tumor cells. On the other hand, passive immunotherapy is composed of preformed elements, whose immediate action on tumor cells is independent of patient's immune system, such as monoclonal antibodies, bispecific antibodies, small molecules and adoptive cell therapy. Antibody-drug conjugates (ADCs) representing antibodies attached to a payload, allowing for a specific delivery of the chemotherapeutic drug, will be considered in this review as part of the passive immunotherapy. Emerging clinical data across both categories demonstrate meaningful anticancer activity.

The aim of this narrative review is to present the most promising recent developments in this field, encompassing diseases usually managed by head and neck cancer tumor boards, that is, not limited to mucosal squamous cell carcinoma of the head and neck (SCCHN), but also including nasopharyngeal carcinoma (NPC), cutaneous squamous cell carcinoma (cSCC), and thyroid cancer. In salivary gland carcinoma, the benefit of ADCs (e.g., trastuzumab deruxtecan) is already known, and research in this field continues.

The present review will focus on the year 2025, which has been particularly successful, with an unprecedented influx of new promising agents and several positive phase III trials. 

**Box 1:**
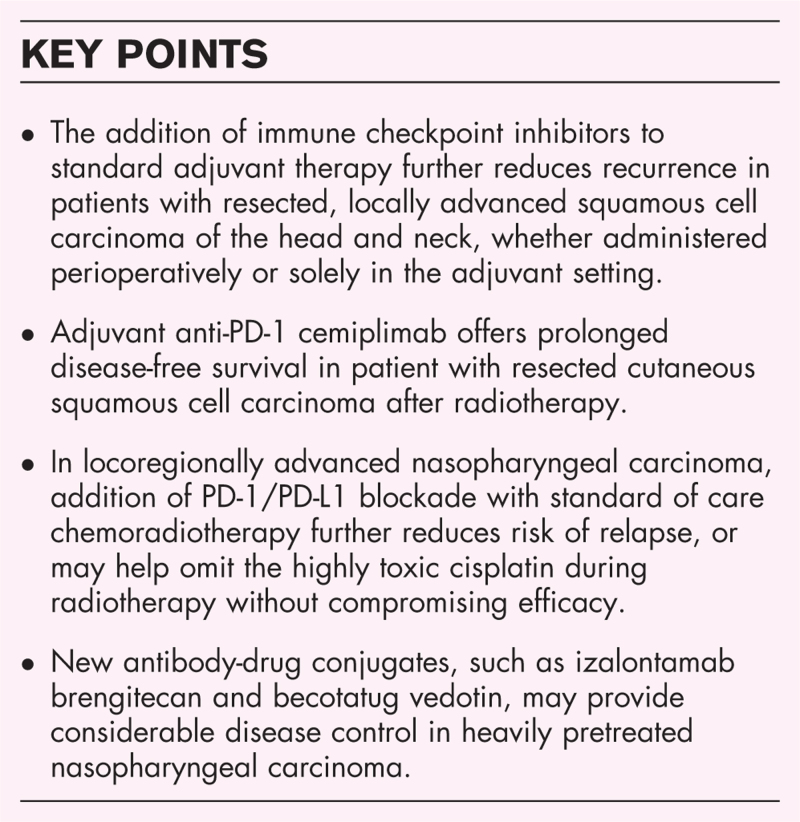
no caption available

## ACTIVE IMMUNOTHERAPY

Studies described in this section investigated the clinical outcome of active immunotherapy agents, mainly ICIs. They are listed in Tables 1 and 2 (Table 1 summarizing the study design characteristics and Table 2 reporting their results).

**Table 1:** Active immunotherapy: study design characteristics

First author	Study name/NCT	Disease entity	Disease setting	Study design	Number of patients (ITT)	Enrolment period	Primary endpoint	Reference
R Uppaluri	KEYNOTE-689	Resectable locally advanced head and neck SCC (stage III–IVA)	Locoregionally advanced (neoadjuvant + adjuvant)	Phase 3	714 (363 pembrolizumab, 351 standard)	2019–2022	PFS	[1^▪▪^]
J Bourhis	NIVOPOSTOP	Completely resected, high-risk^a^ head and neck SCC with high-risk pathological features	Locoregionally advanced (adjuvant)	Phase 3	680, including 666 before data cutoff date (332 (nivolumab) and 334 (standard))	2018–2024	DFS	[2^▪▪^]
YL Liang	DIPPER	Locoregionally advanced NPC	Locoregionally advanced (adjuvant)	Phase 3	450 (226 camrelizumab, 224 control)	2018–2021	EFS	[3]
C Xu	DIAMOND	Locoregionally advanced NPC	Locoregionally advanced (adjuvant)	Phase 3	532 (266 cisplatin-free arm, 266 control)	2021–2022	failure-free survival (noninferiority design) and incidence of all-grade vomiting (superiority design)	[5]
Y Shi	KL167-III-08	Recurrent or metastatic NPC	Advanced/metastatic (1L)	Phase 3	295 (197 Tagitanlimab, 98 control)	2022–2023	PFS	[6]
D Rischin	C-POST	High-risk^b^ postoperative cutaneous SCC (83% in the head and neck)	Adjuvant (curative intent)	Phase 3	415 (209 cemiplimab, 206 placebo)	2019–2024	DFS	[8]
DP Zandberg	Alliance A091802	Recurrent or metastatic cutaneous SCC (84% in the head and neck)	Advanced/metastatic	Phase 2	57 (29 avelumab and cetuximab, 28 avelumab)	2019–2023	PFS	[9]
M Zafereo	NCT04675710	Advanced BRAF V600E-mutated anaplastic thyroid cancer (BRAFm-ATC) (stage IVB/IVC)	Neoadjuvant for advanced stages	Phase 2	42 patients enrolled, 36 included in the reported analysis	2021–2025	R0/R1 resection rate and OS	[11]

DFS, disease-free survival; EFS, event-free survival; NPC, nasopharyngeal carcinoma; OS, overall survival; PFS, progression-free survival; SCC, squamous cell carcinoma. ^a^ High risk (NIVOPOSTOP): any or all of the following: extracapsular extension (ECE) of lymph node, microscopically positive tumor margins (R1 or close margin ≤ 1 mm), ≥ 4 cervical nodal involvements without ECE, multiple peri-neural invasions (PNI). ^b^ High risk (C-POST): nodal features (extracapsular extension with largest node ≥20 mm in diameter or ≥3 involved nodes) or nonnodal features (in-transit metastases, T4 lesion [with bone invasion], perineural invasion, or locally recurrent tumor with ≥1 additional risk feature).

**Table 2:** Active immunotherapy: results

Study name/NCT	Treatment arms	Efficacy	Toxicity and adverse events (all grade, grade ≥3)	Compliance	Median follow-up [months]	Reference
KEYNOTE-689	Arm A: pembrolizumab Q3W (2 neoadjuvant + 15 cycles adjuvant) + adjuvant RT ± cisplatin Q3W 3 cycles (for the high risk^a^) Arm B: adjuvant RT ± cisplatin Q3W 3 cycles (for the high risk^a^)	For combined positive score PD-L1 >1 > median PFS: 59.7 months (arm A) vs. 29.6 months (arm B) (HR = 0.7, *P* = 0.003) > complete pathological response: 9.8% (arm A) vs. 0 (arm B) > 3-year OS: 69.0% (arm A) vs. 60.2% (arm B)	TRAE >All grades: 81.4% (arm A) vs. 81.9% (arm B) >Grade ≥3: 44.6% (arm A) vs. 42.9% (arm B)	>90%	38	[1^▪▪^]
NIVOPOSTOP	Arm A: adjuvant nivolumab Q3W 10 cycles + adjuvant RT + cisplatin Q3W 3 cycles Arm B: adjuvant RT + cisplatin Q3W 3 cycles	> 3-year DFS: 63% (arm A) vs. 52% (arm B) (HR 0.76, *P* = 0.034) > OS immature	TRAE > All grades: 99.3% (arm A) vs. 96.2% (arm B) > Grade≥3: 72.4% (arm A) vs. 67.3% (arm B)	>93%	30.3	[2^▪▪^]
DIPPER	Arm A: Camrelizumab iv Q3W 12 cycles Arm B: observation	> 3-year EFS: 86.9% (arm A) vs. 77.3% (arm B) (HR 0.56, *P* = 0.01) > 3-year OS: 96.4% (arm A) vs. 92.9% (arm B) (not significant)	TRAE > All grades: 97.1% (arm A) vs. 85.5% (arm B) > Grade≥3: 11.2% (arm A) vs. 3.2% (arm B)	81%	39	[3]
DIAMOND	Arm A: toripalimab Q3W 17 cycles + gemcitabine/cisplatin induction chemotherapy, followed by RT (no cisplatin) Arm B: toripalimab Q3W 17 cycles + gemcitabine/cisplatin induction chemotherapy, followed by RT and cisplatin Q3W for 2 cycles	> 3-year failure-free survival 88.3% (arm A) vs. 87.6% (arm B). Difference = 0.7% (*P* = 0.002) > incidence of all-grade vomiting: 26.2% (arm A) vs. 59.8% (arm B). Difference of 33.6% (*P* < 0.001)	All adverse event > All grades: 100% (arm A and B) > Grade≥3: 52.3% (arm A) vs. 63.6% (arm B)	94.7%	37	[5]
KL167-III-08	Arm A: 6 cycles of cisplatin Q3W + gemcitabine (D1 and D8 Q3W) + tagitanlimab Q3W, followed by tagitanlimab Q3W Arm B: 6 cycles of cisplatin Q3W + gemcitabine (D1 and D8 Q3W) + placebo Q3W, followed by placebo Q3W	> median PFS: NR (arm A) vs. 7.9 months (arm B) (HR 0.47, *P* = 0.0001) > 12-month PFS: 56.7% (arm A) vs. 26.7% (arm B) > ORR 81.7% (arm A) vs. 74.5% (arm B) > median DoR: 11.7 vs. 5.8 months > OS immature	As reported in the ESMO presentation TRAE > All grade: 100% (arm A and B) > Grade≥3: 86.3% (arm A) vs. 80.6% (arm B)	NS	11.7	[6]
C-POST	Arm A: adjuvant cemiplimab Q3W 4 cycles, followed by 6 additional cycles Q6W Control arm: adjuvant placebo Q3W 4 cycles, followed by 6 additional cycles Q6W	> median DFS: NR (arm A) vs. 49.9 months (arm B) (HR 0.32, *P* < 0.001) > 24-months DFS: 87.1% (arm A) vs. 64.1% (arm B)	All adverse event > All grades: 91.2% (arm A) vs. 89.2 (arm B) > Grade≥3: 23.9% (arm A) vs. 14.2% (arm B)	≥90%	24	[8]
Alliance A091802	Arm A: avelumab and cetuximab Q2W for 1 year, and avelumab Q2W for up to a second year. Arm B: avelumab alone Q2W for up to 2 years.	> median PFS: 11.1 months (arm A) vs. 3 months (arm B) (HR = 0.48, *P* = 0.018) > ORR: 27.6% (arm A) vs. 21.4% (arm B) > median OS: NR (arm A) vs. 35.8 months (arm B) (HR = 0.78, not significant)	TRAE > All grades: 93% (arm A) vs. 79% (arm B) > Grade≥3: 48% (arm A) vs. 21% (arm B)	69%	34.6	[9]
NCT04675710	Arm A (single-arm): dabrafenib/ trametinib for a 3 to 6-week run-in, followed by addition of pembrolizumab Q3W until surgery, with restating Q3W. In the postoperative period: pembrolizumab ± RT ± dabrafenib/trametinib	> R0/R1 resection rate: 81% > pathologic complete response: 56% > median OS: 20 months	>All grades and grade 1-4 not reported in the abstract >Grade = 5: 22%, including 17% unlikely treatment-related deaths	NS	18	[11]

DFS, disease-free survival; DoR, duration of response; EFS, event-free survival; NR not reached; NS, not specified; OS, overall survival; PFS, progression-free survival; TRAE, treatment-related adverse event; Q2W, every 2 weeks; Q3W, every 3 weeks; Q6W, every 6 weeks; RT, radiotherapy.

^a^ High risk (KEYNOTE-689): presence of positive margins (<1 mm) or extranodal extension.

### Mucosal squamous cell carcinoma

Until 2025, treatment of resectable locoregionally advanced SCCHN was driven by surgery followed by risk-adapted adjuvant therapy based on pathological features of the surgical specimen: postoperative radiotherapy alone for low-risk patients or chemoradiotherapy (CRT) for high-risk patients. Two seminal trials are reshaping the landscape of resectable LA-SCCHN by adding antiprogrammed cell death-1 (PD-1) ICIs to standard therapy, evaluating pembrolizumab [1^▪▪^] and nivolumab [2^▪▪^] in distinct perioperative and adjuvant settings.

KEYNOTE-689 was a large, international phase 3 trial enrolling 714 patients with newly diagnosed, resectable LA-SCCHN (stage III-IVA), including both low-risk and high-risk groups stratified postsurgery. The experimental arm included 17 total doses of pembrolizumab: two neoadjuvant, three concurrent with risk-adapted RT/CRT, and 12 maintenance doses thereafter, compared to upfront surgery followed by risk-adapted adjuvant RT or CRT. Pembrolizumab significantly improved 3-year event-free survival (EFS) with a hazard ratio of 0.70 for the population with a programmed cell death-ligand 1 (PD-L1) expression measured as combined positive score (CPS) of 1 or more, showing durable, early separation of survival curves. The statistical analysis used a nested approach, evaluating the populations with CPS at least 10, then at least 1, and finally the total population. Although positive EFS results were observed across the entire cohort, the small number of patients with PD-L1 negative tumors requires cautious interpretation, and further studies are needed to clarify pembrolizumab's efficacy in this subgroup. Additionally, underrepresentation of human papillomavirus (HPV)-positive patients may limit generalizability to this population. Open questions remain about the relative benefits of adjuvant vs. neoadjuvant pembrolizumab and the clinical significance of major pathological response. Mature overall survival (OS) data are still awaited.

In contrast, the NIVOPOSTOP trial enrolled exclusively high-risk patients after surgery, randomly assigning them to adjuvant nivolumab plus standard CRT vs. CRT alone [2^▪▪^]. Nivolumab was administered as one dose postoperatively prior to radiotherapy, followed by three three-weekly doses concurrent with CRT, and six maintenance monthly doses. The primary endpoint was disease-free survival (DFS). NIVOPOSTOP demonstrated significant DFS improvement (HR similar to KEYNOTE-689) with manageable safety, supporting nivolumab as an effective adjunct to intensive CRT in high-risk LA-SCCHN.

One important difference between the two trials relies on the studied population. Because of its perioperative approach, the KEYNOTE-689 involved resectable LA-SCCHN (III-IVA) patients, including those at a low- or high-risk of recurrence (classified after surgery), whereas the NIVOPOSTOP focused only on high-risk patients. As direct consequence of this, the standard of care adjuvant differed, in addition to ICI, with proportionally less patients receiving cisplatin in the immunotherapy arms in KEYNOTE-689 relative to NIVOPOSTOP, reflecting the low-risk population included in the former study.

Both trials confirm that incorporating anti-PD-1 ICIs perioperatively or as adjuvant therapy reduces recurrence risk and improves survival outcomes compared to historical data. Remarkably, immunotherapy in NIVOPOSTOP mainly reduced locoregional failures, while the perioperative addition of pembrolizumab decreased mostly distant recurrences. In summary, these studies establish a new standard of care for operable LA-HNSCC by demonstrating the clinical benefit of immunotherapy timing and risk-adapted intensity, with both pembrolizumab and nivolumab safely enhancing curative treatment strategies and providing improved long-term outcomes.

### Nasopharyngeal carcinoma

Recent trials have tested ICIs at various stages of NPC treatment. The DIPPER and DIAMOND trials focused on LA-NPC, typically treated with induction chemotherapy containing cisplatin/gemcitabine doublet, followed by cisplatin-based concurrent CRT, while another phase III trial evaluated immunotherapy in the palliative setting.

The DIPPER trial evaluated the addition of adjuvant camrelizumab (anti–PD-1) after definitive CRT. Patients were randomized to receive 12 cycles of camrelizumab or observation [3]. Camrelizumab significantly improved 3-year EFS to 86.9 vs. 77.3% in the control arm. Both locoregional and distant recurrences were curtailed by camrelizumab, with manageable toxicity and no adverse quality-of-life impact. A well known camrelizumab-related adverse event of the skin, called reactive capillary endothelial proliferation, occurred in 88% of patients but was predominantly grade 1–2. Despite the relationship between this adverse effect and prolonged progression-free survival (PFS) in hepatocellular carcinoma [4], such correlations were not observed here. Taked together, this trial suggests benefits of adjuvant camrelizumab in high-risk LA-NPC after definitive CRT.

The DIAMOND study tested whether the omission of concurrent cisplatin during RT could be compensated for by adding toripalimab (anti–PD-1) to induction chemotherapy [5]. All 532 patients first received toripalimab plus gemcitabine and cisplatin induction and were then randomized to RT alone vs. standard concurrent CRT with cisplatin. The 3-year failure-free survival (FFS) was similar (~88%) between groups, meeting noninferiority criteria, while toxicity and nausea were significantly reduced in the cisplatin-sparing arm. Thus, toripalimab allows cisplatin omission during CRT without compromising efficacy.

In the palliative setting, a trial by Shi *et al.* enrolled previously untreated recurrent/metastatic (R/M) NPC [6]. All patients received six cycles of gemcitabine and cisplatin, with the addition of tagitanlimab (anti-PD-L1) or placebo, followed by a maintenance of tagitanlimab or placebo. Despite short median follow-up (11.7 months), tagitanlimab significantly improved PFS (median not reached vs. 7.9 months; hazard ratio 0.47), associated with prolonged duration of response (DoR) and increased overall response rate (ORR), with manageable toxicity. This trial allowed approval of tagitanlimab combined with the gemcitabine and cisplatin regimen as first line treatment for R/M NPC.

These trials collectively demonstrate the efficacy of ICIs in different stages of NPC: adjuvant camrelizumab improves EFS post-CRT in LA-NPC, toripalimab enables cisplatin-sparing CRT with maintained outcomes, and tagitanlimab extends PFS in R/M-NPC. This evidence supports the use of ICIs across NPC disease stages to enhance outcomes while maintaining acceptable toxicity rates.

### Cutaneous squamous cell carcinoma

Cutaneous squamous cell carcinoma is one of the most prevalent cancers worldwide and most commonly arise on sun-exposed areas, including the head and the neck location. Immunotherapy is being explored both after curative treatment and in a more advanced setting. The C-POST trial evaluated adjuvant ICI administration vs. placebo after surgery and postoperative RT in patients with resected, high-risk cSCC, mostly in the head and neck area [7]. Cemiplimab significantly improved DFS (hazard ratio of 0.32), with a reduction of both locoregional and distant recurrences. Toxicity profile was manageable and consistent with known anti-PD-1 side effects. This trial strengthens the role of adjuvant immune checkpoint inhibition after curative-intent treatment, to reduce recurrences.

These findings contrast with the negative KEYNOTE-630 trial in a similar setting of high-risk LA-cSCC [8]. Similarly to the C-POST trial with cemiplimab, this phase 3 study evaluated the clinical benefit of adjuvant pembrolizumab vs. placebo in addition to definitive local treatment. Despite a tendency for prolonged PFS, the results did not meet the threshold for statistical significance. Although the exact explanations for these discrepant results have not been fully elucidated, differences in the studied populations may be implicated. Deeper comparison analyses may help identify subgroups most likely to benefit from adjuvant PD-1 blockade.

For metastatic cSCC, standard first-line systemic treatment strategy relies on cemiplimab monotherapy (anti-PD-1) [9]. Zandberg *et al.* [10] evaluated clinical outcomes of the addition of epidermal growth factor receptor (EGFR) inhibition to the standard blockade of the PD-1/PD-L1 axis. The Alliance phase 2 trial randomized patients with recurrent or metastatic cSCC, predominantly originating in the head and neck region, to receive a combination of avelumab (anti-PD-L1) and cetuximab (anti-EGFR) vs. avelumab alone. Cetuximab is believed to work as an immune-stimulating agent in this setting, synergizing effects with the PD-L1 blockade. The combination significantly improved PFS (hazard ratio 0.48) and showed a tendency to improved OS but was associated with a higher frequence of treatment-related adverse events of grade 3 or more (48 vs. 22%), particularly due to infusion reactions and acneiform rash.

These studies highlight the promising role of ICIs in reducing the recurrence rate in high-risk resected cSCC and in providing longer control of more advanced disease when combined with an anti-EGFR approach, for which therapeutic alternatives remain limited.

### Thyroid cancer

A phase 2 trial evaluated pembrolizumab plus dabrafenib and trametinib in BRAF V600E-mutated anaplastic thyroid cancer (ATC), often remaining unresectable despite dabrafenib and trametinib treatment [11]. The study aimed to convert unresectable disease to resectable status and improve survival. Investigators showed an impressive 74% rate of R0/R1 resections (vs. historical 5%), with 55% complete pathological responses, and a median OS of 18 months. However, grade 5 treatment-related toxicities occurred in 22% (16% being possibly unrelated). Of note, high toxicity has similarly been reported in melanoma with this triplet therapy [12], underscoring the need for cautious risk-benefit assessment. Overall, these data support pembrolizumab plus dabrafenib and trametinib as a promising neoadjuvant approach for BRAF-mutated ATC, with toxicity vigilance.

## PASSIVE IMMUNOTHERAPY

Encouraging studies, using passive immunotherapy agents in the head and neck oncology, are detailed in this section. Their design characteristics are summarized in Table 3, and the results are shown in Table 4.

**Table 3:** Passive immunotherapy: design characteristics

First author	Study name/NCT	Disease entity	Disease setting	Study design	Number of patients (ITT)	Enrolment period	Primary endpoint	Reference
C van Herpen	NCT03526835	Recurrent or metastatic PD-L1-positive head and neck SCC, with no prior systemic therapy in the recurrent or metastatic setting	Advanced/metastatic (1L)	Phase 2 ƒ	45 patients	2018-2024	ORR and safety	[14^▪^]
CH Chung	NCT04429542	Unresectable recurrent or metastatic PD-L1-positive treatment-naive head and neck SCC	Advanced/metastatic (1L)	Phase 1/1b ƒ	Reported in ASCO presentation 40 patients HPV-negative	2020-2024	ORR, DoR, PFS, OS, safety	[16^▪^]
PL Swiecicki	EV-202 (cohort 9)	Recurrent or metastatic PD-L1- positive head and neck SCC (1L)	Advanced/metastatic (1L)	Phase 2	41 patients	2020-2025	ORR	[19]
K Harrington	OrigAMI-4 study, cohort 1	Recurrent or metastatic HPV-unrelated head and neck SCC with ≥1 lines of systemic chemotherapy, including platinum-containing regimen and PD-(L)1 inhibitor	Advanced/metastatic (2L)	Phase 1b/2 ƒ	86 patients enrolled, 38 included in the efficacy population	2024-2025	ORR	[20]
F Han	NCT05126719	Recurrent or metastatic NPC with ≥2 lines of systemic chemotherapy and PD-(L)1 inhibitor	Advanced/metastatic (3L and +)	Phase 2b	173 patients (MRG003 = 86, capecitabine = 36, docetaxel = 51)	2021-2025	ORR, PFS and OS	[21]
Y Yang	BL-B01D1–303	Recurrent or metastatic NPC with ≥2 lines of systemic chemotherapy, including platinum-containing regimen and PD-(L)1 inhibitor	Advanced/metastatic (3L and +)	Phase 3	386 patients (iza-bren = 191 and chemotherapy = 195)	2023-2025	ORR and OS	[22]

DFS, disease-free survival; DoR, duration of response; NPC, nasopharyngeal carcinoma; ORR, objective response rate; OS, overall survival; PFS, progression-free survival; SCC, squamous cell carcinoma; TEAE, treatment-emergent adverse events; TRAE, treatment-related adverse events.

ƒ Ongoing or planned phase 3 trials:

• Petosemtamab: LiGeR-HN1 [15].

• Ficerafusp: FORTIFI-HN01 [17].

• Amivantamab: OrigAMI-5.

**Table 4:** Passive immunotherapy: results

Study Name/NCT	Treatment arms	Efficacy	Toxicity and adverse events (all grade, grade ≥3)	Compliance	Median follow-up [months]	Reference
NCT03526835	Arm A (single arm): Petosemtamab Q2W and pembrolizumab Q6W	> ORR: 60% > median DoR: 11 months > median PFS: 9 months	TEAE > All grade: 100% > Grade ≥3: 60%	NS	14.3	[14^▪^]
NCT04429542	Arm A (single arm): ficerafusp alfa QW + pembrolizumab 200 mg Q3W	For the 40 HPV-negative patients > ORR: 64% > median DoR: 1.4 months > median PFS 9.9 months	TEAE > All grade: 93% > Grade ≥3: 47%	NS	25.2	[16^▪^]
EV-202 (cohort 9)	Arm A (single arm): enfortumab vedotin on D1 and D8 Q#W and Pembrolizumab Q3W	> confirmed ORR 39% > omplete response rate 9.8% > median PFS 5.1 months > median OS and DoR immature	As reported in the ESMO congress in Sept 2025 TRAE > All grade: 92.7% > Grade ≥3: 41.5%	Median number of cycles 6 (for EV and P)	11	[19]
OrigAMI-4 study, cohort 1	Arm A (single arm): subcutaneous amivantamab Q3W	> ORR: 45% > median PFS: 6.8 months > time to first response: 6.4 weeks > median DoR: 7.2 months > median OS: NR	Total TEAE or TREA not specified Among the most common TEAE: > Fatigue: all grade 31%, G≥3 5% > Dermatitis acneiform: all grade 20%, G≥3 7% > Hypoalbuminemia: all grade 31%, G≥3 2%	> 90%	8.3	[20]
NCT05126719	Arm A: MRG003 Q3W Arm B: chemotherapy (capecitabine po or docetaxel iv)	>ORR:30.2% (arm A) vs. 11.5% (arm B). Difference: 18.7%, *P* = 0.0025 > median PFS: 5.8 months (arm A) vs. 2.8 months (arm B) (HR = 0.63, *P* = 0.0146) > median OS: 17.1 months (arm A) vs. 12.0 months (arm B) (HR = 0.73, not significan)	TRAE > All grade: NS > Grade ≥3: 45.3% (arm A) vs. 50.6% (arm B)	NS	13.3	[21]
BL-B01D1–303	ArmA: iza-bren iv D1+D8 Q3W Arm B: chemotherapy (capecitabine po, gemcitabine iv, docetaxel iv)	> ORR: 54.6% (arm A) vs. 27% (arm B). Difference: 27.9%, *P* < 0.0001 > median DoR: 8.5 months (arm A) vs. 4.7 months (arm B) > median PFS: 8.4 months (arm A) vs. 4.3 months (arm B)	TRAE > All grade: 100% (arm A) vs. 95% (arm B) > Grade ≥3: 80% (arm A) vs. 62% (arm B)	> 95%	7.7	[22]

DFS, disease-free survival; DoR, duration of response; EFS, event-free survival; NR, not reached; NS, not specified; OS, overall survival; PFS, progression-free survival; TEAE, treatment-emergent adverse event; TRAE, treatment-related adverse event.

### Mucosal squamous cell carcinoma

Pembrolizumab, given alone or with platinum-fluoropyrimidine chemotherapy, remains standard first-line treatment for R/M-SCCHN based on the KEYNOTE–048 trial, which established pembrolizumab-based regimens as the standard of care in this setting [13].

Building on this backbone, several early–phase studies are exploring bispecific or bifunctional antibodies in combination with PD-1 blockade. Petosemtamab, a first-in-class EGFR × Leucine-Rich Repeat-Containing G-Protein–Coupled Receptor 5 (LGR5) bispecific antibody, combined with pembrolizumab, has shown encouraging response rates (ORR of 60%) and a manageable safety profile as first-line therapy in PD-L1 positive R/M-SCCHN [14^▪^], supporting the ongoing phase 3 LiGeR–HN1 trial (expected to enroll 500 patients) comparing this combination with standard pembrolizumab-based treatment [15].

Chung *et al.* [16^▪^] investigated ficerafusp-alfa, a bifunctional EGFR-targeting and human transforming growth factor beta (TGF-β)-neutralizing antibody. Although the initial phase 1/1b trial enrolled R/M-SCCHN patients, irrespectively of the HPV status, investigators focused on the poor prognosis group of HPV-negative cases, who seem to benefit the most from the ficerafusp/pembrolizumab doublet. In this population, the ORR reached 64%, with durable responses, prolonged PFS and OS (median OS of 21.3 months), and a manageable toxicity profile. These data provide a strong rationale for an ongoing phase 3 FORTIFI-HN01 trial, testing the combination of ficerafusp-alfa and pembrolizumab in HPV-negative R/M-SCCHN [17].

Enfortumab vedotin, a Nectin-4 antibody–drug conjugate, plus pembrolizumab demonstrated strong activity in advanced urothelial cancers [18]. Swiecicki *et al.* [19] sought to evaluate its efficacy as first–line therapy in R/M-SCCHN in the single-arm EV-202 study (cohort 9), with ORR as the primary endpoint. Data reported during the European Society For Medical Oncology (ESMO) Meeting 2025 showed that enfortumab vedotin given together with pembrolizumab was associated with a 39% ORR (irrespectively of the nectin-4 expression) and prolonged PFS (OS and DoR data are immature). The manageable safety profile was consistent with prior reports. These findings demonstrate meaningful synergy between enfortumab vedotin and pembrolizumab in R/M-SCCHN, warranting further randomized evaluation.

In the phase 1b/2 OrigAMI-4 study, investigators aimed at evaluating amivantamab, a bispecific antibody disrupting EGFR and (mesenchymal-epithelial transition factor) MET signaling, in different populations and with various drug combinations. The cohort 1 included HPV-negative R/M-SCCHN patients pretreated with platinum and PD-(L)1 inhibitors, and administered subcutaneous amivantamab monotherapy in the second line (single arm). This regimen was associated with an ORR of 45%, with rapid and durable responses (median DoR of 7.2 months) [20]. The subcutaneous formulation was associated with a low rate of administration-related reactions (7%, all grade 1 or 2) and a generally manageable safety profile.

All these trials are early phase trials with promising results regarding ORR, with some agents offering rapid and durable responses. Because of their single-arm designs, direct comparisons between studies are not possible. Nonetheless, the strength of these preliminary results has prompted the initiation of phase 3 programs, which are already planned or underway for certain agents, including petosemtamab in LiGeR-HN1 [15], ficerafusp-alfa in FORTIFI-HN01 [17], and amivantamab in OrigAMI-5.

### Nasopharyngeal carcinoma

Two pivotal studies evaluate ADC-based strategies in heavily pretreated R/M-NPC, offering new options to a population with poor prognosis.

Becotatug vedotin (MRG003), an EGFR-targeting ADC, showed promising results in a phase 2b randomized trial vs. chemotherapy, after at least two prior lines including platinum and PD-1/L1 inhibitors [21]. MRG003 offered a significantly increased ORR of 30.2 vs. 11.5%, as well as prolonged PFS, reaching 5.82 vs. 2.83 months. The rate of grade at least 3 treatment-related adverse events was slightly lower compared with chemotherapy.

The phase 3 BL-B01D1-303 trial evaluated izalontamab brengitecan (iza-bren), a first-in-class EGFR/human epidermal growth factor receptor 3 (HER3) bispecific ADC, vs. physician's choice chemotherapy in patients with R/M-NPC after at least two prior lines of chemotherapy (including platinum and PD-1/PD-L1 blockade) [22]. Iza-bren achieved superior ORR (54.6 vs. 27.0%), with prolonged DoR and PFS, although this was offset by higher toxicity rates (serious adverse events in 43 vs. 27%). Data about the OS are awaited.

## CONCLUSION

The influx of new drugs will raise important discussions about optimal treatment sequencing and revisiting of the rationale for retreatment or rechallenge strategies, which must consider both the efficacy and toxicity of prior regimens. In this context, a key question will be how to restore sensitivity to immunotherapy, for instance, by intercalating other agents between the first and subsequent immunotherapy lines, or by opting for combination strategies that include components designed to overcome resistance mechanisms to ICIs. Another emerging challenge will be how best to integrate these novel systemic approaches with local treatments, not only in the curative setting, as we have seen with the pivotal KEYNOTE-689 and NIVOPOSTOP trials, but also in the R/M setting, which has already demonstrated feasibility and activity in oligometastatic disease, with promising potential for polymetastatic cases [23].

## Acknowledgements

*None.*

### Financial support and sponsorship

*None.*

### Conflicts of interest

*Petr Szturz reports institutional relationships with Merck KGaA (speakers’ bureau, advisory board), Servier (consulting, advisory board, speakers’ bureau), Takeda Pharma AG (consulting), and Merck Sharp & Dohme Corp. (consulting, advisory board).*

## REFERENCES AND RECOMMENDED READING

Papers of particular interest, published within the annual period of review, have been highlighted as:

▪ of special interest
▪▪ of outstanding interest
